# Intermittent Ileitis and Partial Small-Bowel Obstruction Due to Intestinal Helminth Infection Mimicking Crohn’s Disease

**DOI:** 10.7759/cureus.111038

**Published:** 2026-06-17

**Authors:** Lucas Youno, Caddie Nguyen, Sabreen Hamad, Jackelyn Philip, Michael Herman

**Affiliations:** 1 Gastroenterology, Lincoln Memorial University-DeBusk College of Osteopathic Medicine, Jacksonville, USA; 2 Dermatology/Gastroenterology, Lincoln Memorial University-DeBusk College of Osteopathic Medicine, Jacksonville, USA; 3 Gastroenterology, Borland Groover, Jacksonville, USA

**Keywords:** ascariasis, ascaris lumbricoides, colonoscopy, crohn’s disease mimic, eosinophilia, helminth infection, intestinal nematode, parasitic infection, small-bowel obstruction, terminal ileitis

## Abstract

Intestinal helminth infections may present with chronic gastrointestinal symptoms and can mimic inflammatory or obstructive bowel pathology, leading to diagnostic challenges. A 27-year-old previously healthy male presented with nine months of intermittent abdominal pain, nausea, and bloating with concern for partial small-bowel obstruction. He reported frequent hunting and environmental exposure associated with field dressing and preparation of wild game. Imaging demonstrated distal ileal wall thickening with upstream dilatation, and laboratory studies revealed marked peripheral eosinophilia. Despite negative stool ova and parasite testing performed after endoscopic retrieval of the parasite, colonoscopy identified a live helminth in the terminal ileum, which was retrieved endoscopically. Histopathologic evaluation demonstrated an intestinal nematode most compatible with *Ascaris lumbricoides*.​​​​​ Treatment with a single 400-mg oral dose of albendazole resulted in rapid symptom resolution and sustained clinical improvement on follow-up. This case emphasizes the importance of obtaining a detailed exposure history and maintaining suspicion for parasitic infection in patients with chronic gastrointestinal symptoms, eosinophilia, and small-bowel inflammation, even when stool studies are negative.

## Introduction

Intestinal helminth infections are rare in developed countries but remain an underrecognized cause of gastrointestinal disease [1-3]. Clinical manifestations range from asymptomatic infection to chronic intermittent abdominal pain, ileitis, and episodic obstruction and may clinically resemble Crohn's disease or other inflammatory bowel conditions [4,5]. These presentations often lead to extensive diagnostic evaluation, including cross-sectional imaging, before the underlying etiology is identified [6,7].

Helminthic infections may be underrecognized because symptoms are frequently nonspecific, imaging findings may mimic inflammatory bowel disease, clinical suspicion is often low in nonendemic settings, and stool ova and parasite testing may demonstrate limited sensitivity in low-burden infections or during periods of absent egg shedding.

Diagnosis is further complicated by the limited sensitivity of stool ova and parasite testing, particularly in low-burden infections or when adult worms are present without active egg shedding [3,4]. Environmental and soil exposure may increase susceptibility to certain helminth infections. In this case, hunting-related activities and environmental exposure were considered epidemiologically relevant; however, the specific source of infection could not be definitively established [2]. We present a case illustrating these diagnostic challenges and the critical role of endoscopic visualization.

## Case presentation

A 27-year-old male with no significant past medical or surgical history presented with acute abdominal pain, intractable nausea, and vomiting. He reported that symptoms had been intermittent for approximately nine months, consisting of episodic epigastric and periumbilical pain, bloating, early satiety, and nausea. Episodes occurred unpredictably and resolved spontaneously.

He denied weight loss, fevers, night sweats, diarrhea, hematochezia, melena, dysphagia, or anorexia. Bowel movements were normal between episodes. He took no prescription or over-the-counter medications and denied alcohol, tobacco, or illicit drug use. Family history was notable for a brother with ulcerative colitis.

Social history was significant for avid hunting and frequent participation in field dressing and preparation of locally harvested game. He denied recent international travel.

On presentation, abdominal pain intensified and was associated with multiple episodes of emesis and radiation to the back. Physical examination demonstrated mild diffuse abdominal tenderness without peritoneal signs. Laboratory evaluation demonstrated mild leukocytosis with marked peripheral eosinophilia, a key finding that broadened the differential diagnosis to include parasitic infection, while hemoglobin, renal function, liver function tests, and inflammatory markers remained within normal limits (Table 1).

**Table 1:** Laboratory findings at the initial presentation demonstrating marked peripheral eosinophilia. AST: Aspartate aminotransferase; ALT: alanine transaminase

Laboratory Test	Result	Reference Range	Units
White Blood Cell Count	12.4	4.0–11.0	×10³/µL
Hemoglobin	15.1	13.5–17.5	g/dL
Hematocrit	44.8	41–53	%
Platelet Count	287	150–400	×10³/µL
Absolute Eosinophil Count	3.6	0.0–0.5	×10³/µL
Eosinophils	29	0–6	%
Sodium	138	135–145	mmol/L
Potassium	4.2	3.5–5.1	mmol/L
Chloride	102	98–107	mmol/L
Bicarbonate	25	22–29	mmol/L
Blood Urea Nitrogen	14	7–20	mg/dL
Creatinine	0.9	0.7–1.3	mg/dL
AST	24	10–40	U/L
ALT	28	7–56	U/L
C-Reactive Protein	0.4	<0.8	mg/dL
Erythrocyte Sedimentation Rate	8	0–15	mm/hr

CT abdomen and pelvis demonstrated moderate circumferential distal small-bowel wall thickening with upstream dilatation (Figure 1), raising concern for partial small-bowel obstruction. Symptoms improved following a small-bowel follow-through, and he was discharged with outpatient gastroenterology follow-up.

**Figure 1:**
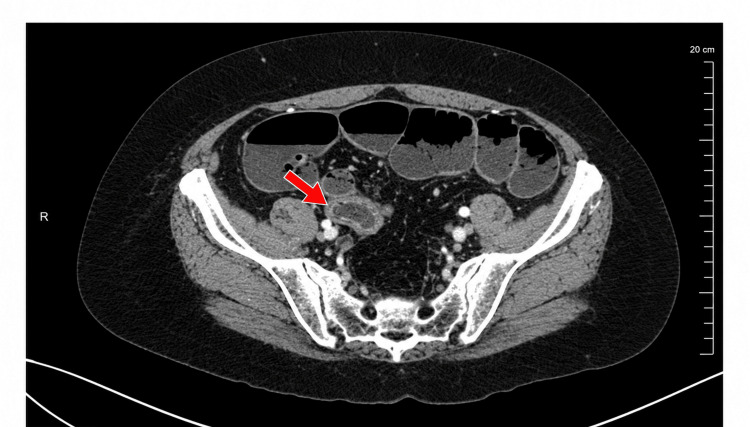
Axial CT image demonstrating circumferential distal ileal wall thickening with upstream small-bowel dilatation, raising concern for partial small-bowel obstruction.

Further evaluation included esophagogastroduodenoscopy (EGD) and colonoscopy on Day 51. EGD revealed a 3-cm sliding hiatal hernia, with an otherwise normal stomach and duodenum. Duodenal biopsies demonstrated mild acute and chronic duodenitis, negative for celiac disease. Gastric biopsies were benign and negative for Helicobacter pylori. Esophageal biopsies were negative for eosinophilic esophagitis.

Colonoscopy revealed a live helminth in the terminal ileum (Figures 2, 3), which was retrieved with biopsy forceps and submitted for pathologic evaluation. Endoscopically, the parasite appeared as a motile, elongated nematode within the terminal ileum. Exact measurements were not formally recorded at the time of retrieval. The remainder of the terminal ileum and colon appeared normal, with biopsies negative for inflammatory bowel disease.

**Figure 2:**
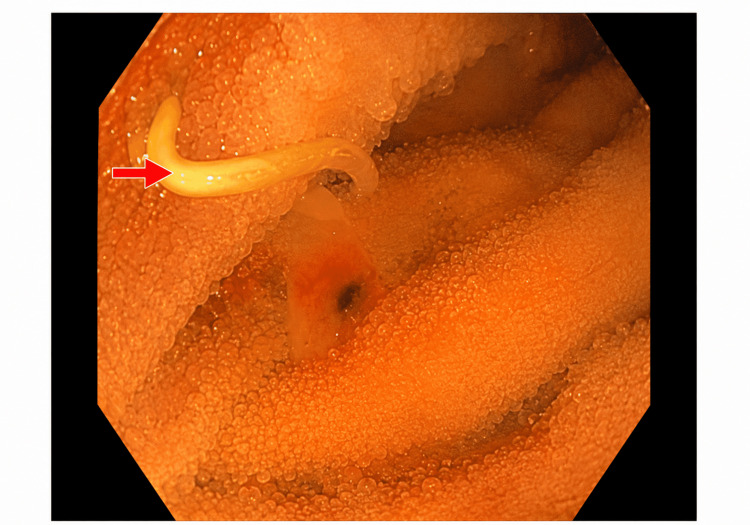
Colonoscopic image demonstrating a live motile helminth within the terminal ileum identified during evaluation of chronic abdominal pain and peripheral eosinophilia.

**Figure 3:**
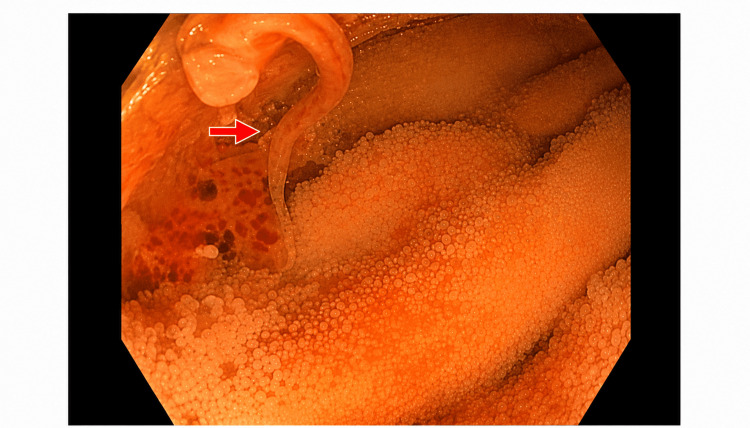
Additional colonoscopic view of the live terminal ileal helminth prior to endoscopic retrieval.

Histopathologic examination demonstrated a cross-section of an intestinal nematode with identifiable intestinal structures, a muscular body wall, and reproductive organs containing numerous ova (Figures 4, 5). The surrounding ileal mucosa demonstrated an eosinophil-rich inflammatory infiltrate. Based on the morphologic findings, anatomic location, peripheral eosinophilia, exposure history, and clinical course, the parasite was most compatible with *Ascaris lumbricoides*. Species-level identification remained presumptive because molecular confirmation was not performed.

**Figure 4:**
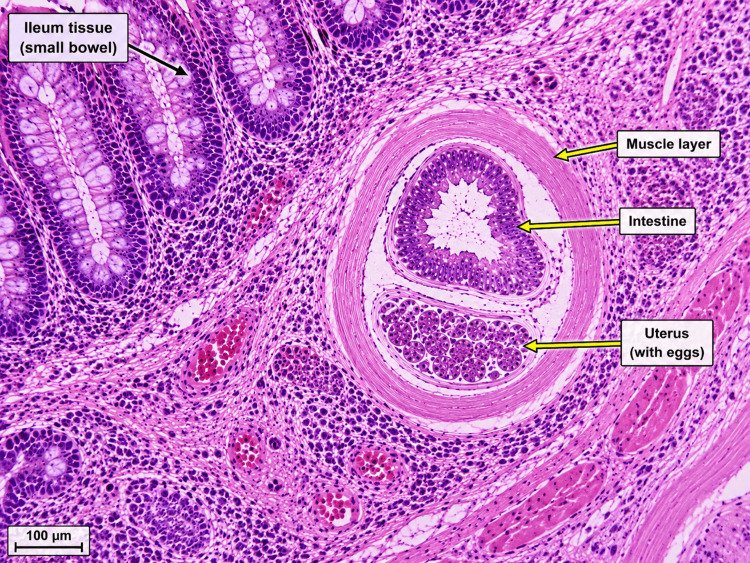
Histopathologic cross-section of an intestinal nematode demonstrating characteristic internal structures and surrounding eosinophil-rich inflammatory infiltrate within terminal ileal tissue.

**Figure 5:**
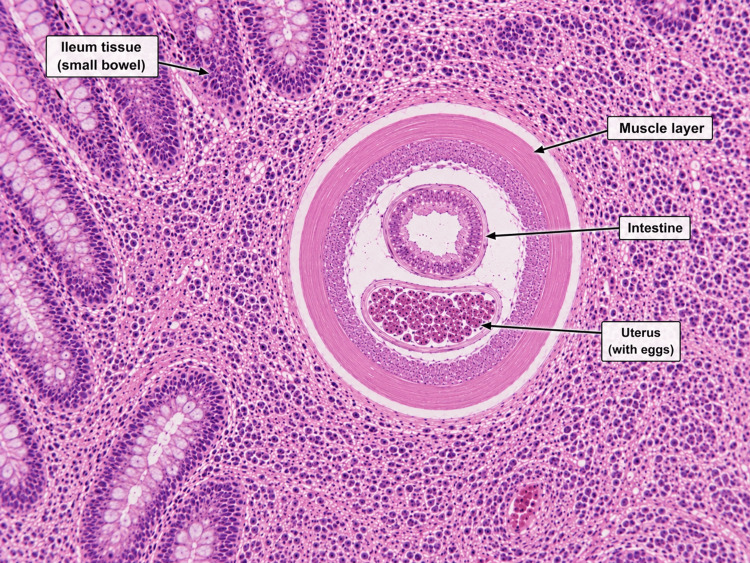
Higher-magnification histopathologic image demonstrating nematode morphology, including intestinal structures and reproductive organs containing numerous ova.

Stool ova and parasite testing performed on Day 53, two days after endoscopic retrieval of the parasite, was negative. The timing of testing may have reduced diagnostic yield. At follow-up, the patient reported complete resolution of abdominal pain, nausea, and bloating without symptom recurrence after treatment with a single 400-mg oral dose of albendazole.

## Discussion

This case illustrates the diagnostic complexity of chronic intermittent small-bowel symptoms in an otherwise healthy young adult and highlights the importance of maintaining a broad differential diagnosis throughout the evaluation. The patient’s nine-month history of episodic abdominal pain and nausea, followed by acute hospitalization with imaging concerning partial obstruction, initially raised concern for Crohn’s disease, eosinophilic gastrointestinal disease, infectious ileitis, parasitic infection, and intermittent mechanical obstruction.

The initial CT findings of distal ileal wall thickening with upstream dilatation are frequently associated with Crohn’s disease, particularly in young patients with recurrent symptoms [1,2]. However, several features made this diagnosis less likely, including the absence of chronic diarrhea, weight loss, perianal disease, or systemic inflammatory markers, as well as normal terminal ileal and colonic biopsies on colonoscopy. Additionally, the intermittent nature of symptoms over many months was atypical for untreated Crohn’s disease.

The presence of marked peripheral eosinophilia served as a key diagnostic clue. Eosinophilia in the setting of gastrointestinal symptoms raises consideration for parasitic infection, eosinophilic gastrointestinal disorders, drug reactions, and, less commonly, malignancy [3]. Eosinophilic gastroenteritis was considered; however, mucosal biopsies did not demonstrate eosinophilic infiltration, and the rapid response to antiparasitic therapy argued against a primary eosinophilic disorder. Nevertheless, mucosal biopsies alone cannot completely exclude eosinophilic gastrointestinal disease because deeper bowel wall involvement may not be sampled endoscopically.

Additional diagnostic considerations included anisakiasis, strongyloidiasis, eosinophilic enteritis, infectious ileitis, and other intestinal helminth infections. These entities were considered, given the patient's eosinophilia and imaging findings but were felt to be less likely following direct endoscopic visualization and histopathologic evaluation of the parasite.

A major diagnostic challenge in this case was the negative stool ova and parasite testing, which could have falsely reassured clinicians. Stool studies are known to have limited sensitivity, particularly in low-burden helminth infections or when adult worms are present without active egg shedding [4-6]. Ascaris infection, in particular, may yield negative stool studies during certain phases of infection or when intestinal involvement is isolated [6]. Additionally, stool testing was performed after endoscopic retrieval of the parasite, which may have further reduced diagnostic sensitivity.

The patient's exposure history was considered epidemiologically relevant but not diagnostic. Although hunting and wild-game exposure prompted consideration of parasitic infection, ingestion of wild game is not a well-established transmission route for *Ascaris lumbricoides*. Rather, hunting activities, field dressing, food preparation, and environmental soil exposure may increase opportunities for contact with contaminated material [8]. Therefore, this history should be interpreted as supportive rather than definitive evidence regarding the source of infection.

Endoscopic evaluation ultimately proved diagnostic. Direct visualization and retrieval of a live worm from the terminal ileum provided definitive evidence of intestinal helminth infection, with histopathologic findings most consistent with *Ascaris lumbricoides*, and avoided unnecessary escalation to immunosuppressive therapy. Multiple reports have described helminth infections mimicking Crohn's disease both clinically and radiographically, reinforcing the importance of excluding infection prior to initiating biologic or steroid therapy [9].

The patient was treated with a single 400-mg oral dose of albendazole and experienced complete symptom resolution within seven days. Albendazole is highly effective against intestinal nematodes, including *Ascaris* species, and symptom improvement within days is well documented [6,10]. The patient's sustained clinical improvement following antihelminthic therapy further supported the diagnosis and argued against inflammatory or structural pathology as the primary etiology.

## Conclusions

This case highlights the importance of obtaining a detailed exposure history and considering parasitic infection in patients with chronic intermittent gastrointestinal symptoms, eosinophilia, and small-bowel inflammation, even in the setting of negative stool studies. Clinicians should maintain a high index of suspicion for helminthic infection in patients with unexplained eosinophilia and obstructive symptoms to avoid misdiagnosis and unnecessary invasive interventions. Prompt recognition can facilitate timely diagnosis and simple, effective antihelminthic therapy while preventing inappropriate escalation to immunosuppressive treatment.
